# Cadmium uptake and partitioning in durum wheat during grain filling

**DOI:** 10.1186/1471-2229-13-103

**Published:** 2013-07-16

**Authors:** Neil S Harris, Gregory J Taylor

**Affiliations:** 1 Department of Biological Sciences, University of Alberta, Edmonton AB T6G 2E9, Canada

**Keywords:** Cadmium (Cd), Durum wheat, Near-isogenic lines (NIL), Grain filling, Uptake, Translocation, Remobilization

## Abstract

**Background:**

Concentrations of cadmium (Cd) in the grain of many durum wheats (*Triticum turgidum* subsp. *durum*) grown in North American prairie soils often exceed international trade standards. Genotypic differences in root-to-shoot translocation of Cd are a major determinant of intraspecific variation in the accumulation of Cd in grain. We tested the extent to which changes in whole-plant Cd accumulation and the distribution of Cd between tissues influences Cd accumulation in grain by measuring Cd accumulation throughout the grain filling period in two near-isogenic lines (NILs) of durum wheat that differ in grain Cd accumulation.

**Results:**

Roots absorbed Cd and transported it to the shoots throughout the grain filling period, but the low- and high-Cd NILs did not differ in whole-plant Cd uptake. Although the majority of Cd accumulation was retained in the roots, the low- and high-Cd NILs differed substantively in root-to-shoot translocation of Cd. At grain maturity, accumulation of Cd in the shoots was 13% (low-Cd NIL) or 37% (high-Cd NIL) of whole-plant Cd accumulation. Accumulation of Cd in all shoot tissue, including grain, was at least 2-fold greater in the high-Cd NIL at all harvests. There was no net remobilization of shoot Cd pools during grain filling. The timing of Cd accumulation in grain was positively correlated with grain biomass accumulation, and the rate of grain filling peaked between 14 and 28 days post-anthesis, when both NILs accumulated 60% of total grain biomass and 61-66% of total grain Cd content.

**Conclusions:**

These results show that genotypic variation in root-to-shoot translocation of Cd controls accumulation of Cd in durum wheat grain. Continued uptake of Cd by roots and the absence of net remobilization of Cd from leaves during grain filling support a direct pathway of Cd transport from roots to grain via xylem-to-phloem transfer in the stem.

## Background

Cadmium (Cd) is a toxic, non-essential element that is naturally present in most soils. While concentrations are typically low (trace), anthropogenic inputs have elevated Cd concentrations in some agricultural soils [1,2]. Cadmium is readily absorbed by roots and transported to above ground portions, including grains [1,2]. Consequently, contaminated foods have become the dominant source of human exposure to environmental Cd [3], with cereals contributing the majority of dietary Cd [2,3]. Some cereals, including rice (*Oryza sativa* L.) and durum wheat (*Triticum turgidum* L. subsp. *durum* (Desf.) Husn.), can accumulate Cd in grain to levels that exceed international trade standards. For example, concentrations of Cd in the grain of many durum wheats grown on the North American prairies [4,5] often exceed the maximum level for Cd in wheat grain (0.2 mg kg^-1^) established by Codex Alimentarius [6].

Cadmium accumulation by plants is influenced by many factors, including available Cd in the soil, soil type and chemistry, climate, agronomic practices, and plant genotype [1,2]. Among the management practices proposed to limit accumulation of Cd in crops [2], breeding for low Cd accumulation has been cited as the most reliable approach [7]. Understanding the mechanisms responsible for genotypic variation in Cd accumulation in grain will accelerate breeding efforts [7,8]. This is particularly true for crops where accumulation of Cd is controlled by major quantitative trait loci (QTL) such as durum wheat, where a single locus (*Cdu1* on chromosome 5B) accounts for 80-90% of phenotypic variation in grain Cd [9-11]. Identification of the genetic factor(s) responsible for *Cdu1* will enable selection of low-Cd genotypes from durum wheat germplasm with different genetic backgrounds without phenotyping or revalidating the marker-*Cdu1* allele relationship [12].

Genotypic differences in root-to-shoot translocation of Cd are a major determinant of intraspecific variation in the accumulation of Cd in grain [3]. As cereals retain the majority of absorbed Cd in the roots [13] variation in translocation of this pool can greatly affect Cd levels in shoot and grain. For example, Uraguchi *et al.*[14] conducted a study on uptake and translocation of Cd by *indica* (high grain Cd) and *japonica* (low grain Cd) cultivars of rice. Although the *japonica* cultivar had greater short-term Cd absorption in roots of seedlings, the high-Cd *indica* cultivar had ≥2-fold greater accumulation in shoots and grain. Furthermore, concentrations of Cd in xylem sap were strongly correlated (*r* = 0.98) with concentrations in shoots [14]. A subsequent screen of 69 diverse cultivars from the world rice collection showed that Cd concentrations in xylem sap were also positively correlated with genotypic variation in grain Cd concentration [14]. Restricted root-to-shoot Cd translocation has also been reported in studies using well-defined genetic systems in which the inheritance of the low-Cd trait is quantitative [15-17].

Differences in Cd accumulation in the grain of durum wheat cultivars and near-isogenic lines (NILs) have also been attributed to genotypic differences in root-to-shoot Cd translocation [18-24]. Differential accumulation of Cd in grain was unrelated to short-term uptake of Cd by roots of seedlings [19,23,25] or maturing plants [21]. Partitioning of Cd between roots and shoots prior to flowering was predictive of Cd accumulation in the grain [8,18,19,21-23,26]. In addition, the Cd concentration of grain was positively correlated with Cd accumulation in other shoot tissues during grain filling [20,22].

Cadmium transported to the shoots of cereals prior to anthesis accumulates in the leaves and stems in a declining gradient towards the developing spike [20,27,28]. Thus, there are multiple pools of Cd (multiple shoot organs and the roots) that are potentially available for remobilization to the grain. Plants can also continue to absorb Cd from the soil during grain filling, directly transporting Cd to the grain via the stem. Unfortunately, evidence for the relative contribution of different pools of Cd (i.e. roots, stems, and leaves) to Cd accumulation in the grain, and for the timing and pathway of Cd transport to the grain is contradictory. Kashiwagi *et al.*[29] documented accumulation of Cd in shoots of field-grown rice prior to and following heading. Loss of Cd from leaves between heading and maturity coincided with increased accumulation of Cd in grain, while shoot Cd content did not change. They concluded that Cd remobilized from the leaves was the primary source of Cd accumulated in the grain [29]. Conversely, rice grown in hydroponic culture continued to absorb Cd from the nutrient solution and translocate it to the shoots during grain filling [30]. Comparison of rice plants supplied with Cd prior to (but not following) flowering with plants supplied Cd only after flowering showed that 40% of grain Cd content was attributable to Cd uptake during grain filling [30].

In durum wheat grown in hydroponic culture, short-term (24 h) uptake of ^106^Cd by roots occurred at tillering, flowering, and grain ripening, while transport of absorbed ^106^Cd to shoots and spikes was reduced at flowering and abolished during ripening [21]. The authors concluded that import of Cd into the spikes was the result of remobilization of Cd from the shoot. In contrast, low- and high-Cd NILs grown in hydroponic culture continued to accumulate Cd in the flag leaf and spikes throughout grain filling [22], and the magnitude of difference between NILs was consistent with differences in root-to-shoot translocation [22,23]. Several studies have interpreted differences in Cd accumulation ratios within shoot organs (e.g. flag leaf:grain Cd concentration ratio) as evidence of remobilization of Cd from vegetative shoot organs to the grain [20,28].

Although some of these differences might be attributed to variations in experimental conditions and the plant genotypes selected, many of the datasets are incomplete. Frequently, only selected tissues are harvested [20,22,29] or shoot tissues are treated as homogenous units (e.g. combining leaf and stems tissues [21,26]). In many experiments grain was harvested at only one time point, typically maturity [20-22,26,27,29]. As a result, these studies do not provide information on the timing of Cd transport to the grain. In a notable exception, Rodda and Reid [30] showed that accumulation of Cd in rice grain occurred during early grain filling (0 to 16 days post-anthesis), coinciding with a period of rapid accumulation of grain biomass. However, Cd accumulation in grain could not be related to whole-plant Cd accumulation or Cd distribution between tissues during grain filling since the experimental design relied on repeated subsampling of single panicles from individual plants [30].

To provide a more thorough understanding of the timing of Cd accumulation in the grain, and to determine if the timing of Cd transport is related to changes in whole-plant Cd accumulation and the distribution of Cd between tissues, durum wheat was grown to maturity in chelator-buffered solution culture. Chelator-buffered solution culture ensures that the roots are exposed to non-toxic, agriculturally-relevant concentrations of Cd. Whole-plants were harvested at first flowering (anthesis) and at different stages during grain filling. Two near-isogenic lines (NILs) of durum wheat differing in accumulation of Cd in grain were compared to determine if changes in accumulation and partitioning during grain filling could account for the observed differences in grain Cd concentration.

## Results

### Growth and cadmium accumulation by seedlings

Seedlings were harvested 21 d after germination when plants had developed 3 or 4 tillers. No differences in growth (dry weight), or whole-plant Cd content between the low- and high-Cd NILs were observed (Table 1). In contrast, NILs differed in distribution of Cd between shoots and roots. The low-Cd NIL retained more Cd in the roots and transported less Cd to the shoots than the high-Cd NIL (Table 1). As a result, the shoot/root Cd content ratio was 2-fold greater in the high-Cd NIL, and the high-Cd NIL displayed lower concentrations of Cd (μg g^-1^) in roots and higher concentrations of Cd in shoots. Accumulation and distribution of the micronutrients, copper (Cu), iron (Fe), manganese (Mn) and zinc (Zn), were not significantly different between the low- and high-Cd NILs (Additional file 1).

**Table 1:** Growth and cadmium accumulation by durum wheat seedlings

	**Tissue**	**Low-Cd**		**High-Cd**
Dry weight (g plant^-1^)	Whole plant	0.72 (0.02)		0.70 (0.03)
	Shoot	0.52 (0.02)		0.49 (0.02)
	Root	0.20 (0.01)		0.21 (0.02)
Cd content (μg plant^-1^)	Whole plant	1.19 (0.05)		1.24 (0.07)
	Shoot	0.29 (0.02)	***	0.51 (0.03)
	Root	0.90 (0.03)	*	0.73 (0.05)
Cd concentration (μg g^-1^)	Whole plant	1.64 (0.03)	*	1.78 (0.04)
	Shoot	0.55 (0.01)	***	1.04 (0.02)
	Root	4.51 (0.16)	***	3.54 (0.07)

Dry weight, Cd content, and Cd concentration of seedlings of low- and high-Cd near-isogenic lines of durum wheat (*Triticum turgidum* subsp. *durum*) grown for 21 d in chelator-buffered nutrient culture containing 0.5 μM Cd (0.014 nM free activity). Significant differences between near-isogenic lines as determined by ANOVA (*F*-test) are indicated by * (*P* ≤ 0.05) and *** (*P* ≤ 0.001). Numbers in parenthesis are SE (*n* = 5).

### Whole-plant growth and cadmium accumulation during grain filling

**Figure 1:**
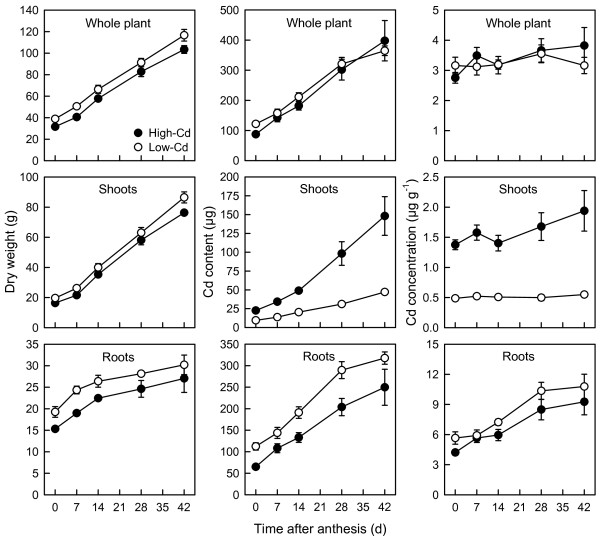
**Whole-plant growth and accumulation of cadmium by durum wheat during grain filling.** Dry weight (whole plant, shoot, root) and Cd (content, concentration) accumulation by low- (open circles) and high-Cd (closed circles) near-isogenic lines of durum wheat (*Triticum turgidum* subsp. *durum*) between anthesis and 42 d post-anthesis. Plants were grown in chelator-buffered nutrient culture containing 0.5 μM Cd (0.014 nM free activity). Plotted values are means ± SE (*n* = 4 or 5).

Near-isogenic lines (NILs) differed (*P* < 0.001) in time to anthesis (high-Cd, 53.7 ± 2.6 d; low-Cd, 58.5 ± 3.3 d). At anthesis, all plants appeared vigorous and free of senescence. By the final harvest (42 days post-anthesis; DPA) symptoms (leaf yellowing) of senescence had begun to appear in the oldest leaves, while younger tissue remained vigorous. Consistent with the longer pre-anthesis growth period, the low-Cd NIL accumulated significantly more shoot, root, and whole-plant biomass than the high-Cd NIL at all harvests (*P* < 0.001; Figure 1). Whole-plant biomass increased linearly during the post-anthesis period (Figure 1), increasing 3-fold between 0 and 42 DPA. Shoot growth accounted for 85% of whole-plant biomass accumulated post-anthesis (NILs combined).

Similar to the seedlings (Table 1), the NILs did not differ in whole-plant Cd accumulation during grain filling, but differed substantively in the distribution of Cd between shoots and roots. Whole-plant Cd content increased 3.6-fold between 0 and 42 DPA (NILs combined; Figure 1). Although whole-plant Cd content of the low-Cd NIL was 6% greater (across all harvests) than the high-Cd NIL (*P* < 0.05), this was attributable to 15% greater biomass in the low-Cd NIL. In contrast, whole-plant Cd concentration did not change during grain filling and did not differ significantly between low- and high-Cd NILs (*P* > 0.05; Figure 1). The Cd content of shoots and roots increased linearly during the post-anthesis period, increasing by 6 and 3.2-fold, respectively (NILs combined) between 0 and 42 DPA. The low-Cd NIL retained more Cd in the roots and transported less Cd to the shoots than the high-Cd NIL. Both the content and concentration of Cd in shoots of the high-Cd NIL were at least 2-fold greater than in low-Cd NIL at all harvests (*P* < 0.001; Figure 1). The content and concentration of Cd in roots of the low-Cd NIL were also greater than in the high-Cd NIL (*P* < 0.01).

### Grain development and cadmium accumulation by grain

**Figure 2:**
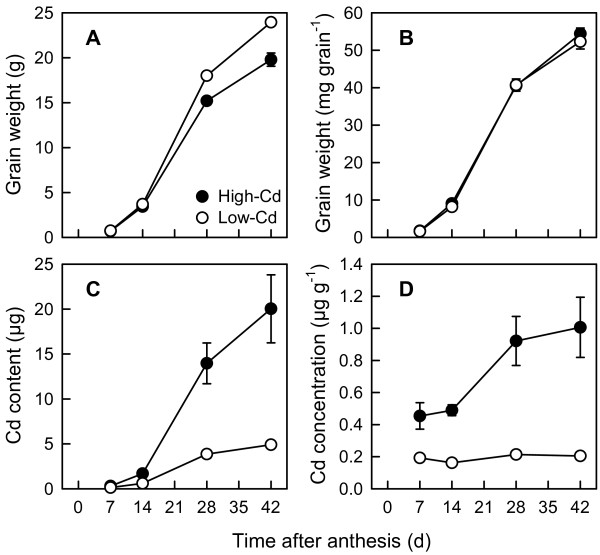
**Grain development and accumulation of cadmium in grain of durum wheat.** Total grain dry weight **(A)**, per grain dry weight **(B)**, grain Cd content **(C)**, and grain Cd concentration **(D)** of low- (open circles) and high-Cd (closed circles) near-isogenic lines of durum wheat (*Triticum turgidum* subsp. *durum*) between anthesis and 42 d post-anthesis. Plants were grown in chelator-buffered nutrient culture containing 0.5 μM Cd (0.014 nM free activity). Plotted values are means ± SE (*n* = 4 or 5).

Grain biomass increased slowly from zero biomass (at anthesis) during the first 7 DPA, and subsequently increased (7–28 DPA) before slowing again as grain approached physiological maturity (28–42 DPA; Figure 2A). The highest rate of grain filling was between 14 and 28 DPA, a period in which both NILs accumulated 60% of total grain DW. Grain development accounted for 33% and 36% (high- and low-Cd NILs respectively) of the biomass accumulated by shoots during the post-anthesis period. Total grain weight of the low-Cd NIL was 19% greater than the high-Cd NIL (*P* < 0.05), a difference that was attributable to the larger heads (number of grain per head) produced. When averaged across all harvests, the low-Cd NIL produced 19 more grain per head than the high-Cd NIL (high-Cd, 97.5 ± 5.7; low-Cd, 116.0 ± 9.5; *P* < 0.001). Near-isogenic lines (NILs) did not differ in dry weight accumulation on a per grain basis (mg grain^-1^) during grain filling (Figure 2B).

Grain Cd content increased during the post-anthesis period in both NILs (Figure 2C), closely paralleling the increase in grain DW. Increasing grain Cd content was highly correlated with DW accumulation (mg grain^-1^) in the low- and high-Cd NILs (*r* = 0.98 and 0.91, respectively). Between 61% (high-Cd) and 66% (low-Cd) of total grain Cd content accumulated between 14 and 28 DPA. However, grain Cd content of the high-Cd NIL was significantly greater than that of the low-Cd NIL at all harvests (*P* < 0.001). Grain Cd concentration of the high-Cd NIL was also greater than that of the low-Cd NIL at all harvests (*P* < 0.001), increasing from 2.4-fold greater Cd concentration at 7 DPA to 4.9-fold at 42 DPA (Figure 2D). Coinciding with the period of rapid grain biomass accumulation, grain Cd concentration increased by 0.4 μg g^-1^ (88%) between 14 and 28 DPA (*P* < 0.05) in the high-Cd NIL. In contrast, grain Cd concentration in the low-Cd NIL did not change over the post-anthesis period (*P* > 0.05), remaining at around 0.2 μg g^-1^.

### Growth and cadmium accumulation of shoot tissues during grain filling

**Figure 3:**
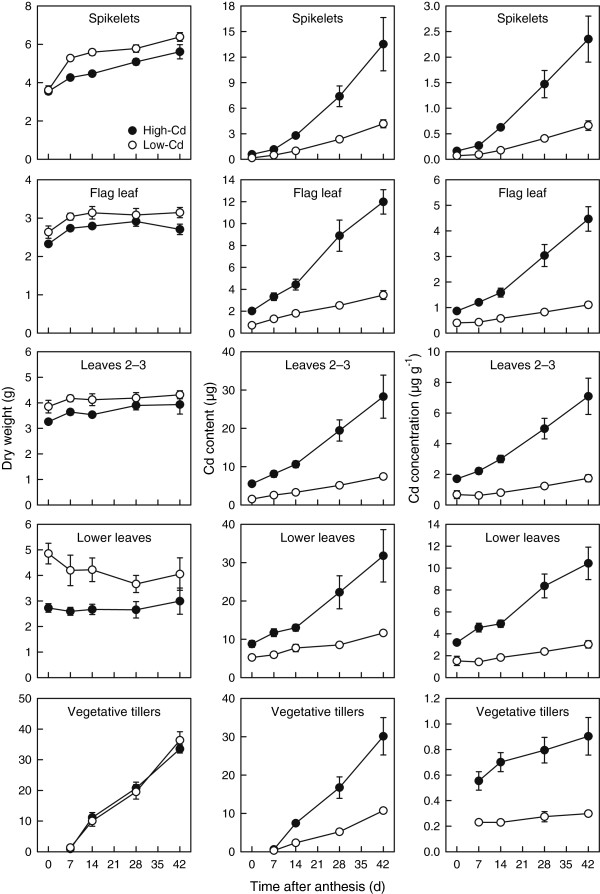
**Growth and cadmium accumulation of spikelets, leaves, and vegetative tillers in durum wheat during grain filling.** Total dry weight, Cd content, and Cd concentration of spikelets, flag leaf, leaves 2–3, lower leaves, and vegetative tillers of low- (open circles) and high-Cd (closed circles) near-isogenic lines of durum wheat (*Triticum turgidum* subsp. *durum*) between anthesis and 42 d post-anthesis. Plants were grown in chelator-buffered nutrient culture containing 0.5 μM Cd (0.014 nM free activity). Plotted values are means ± SE (*n* = 4 or 5).

**Figure 4:**
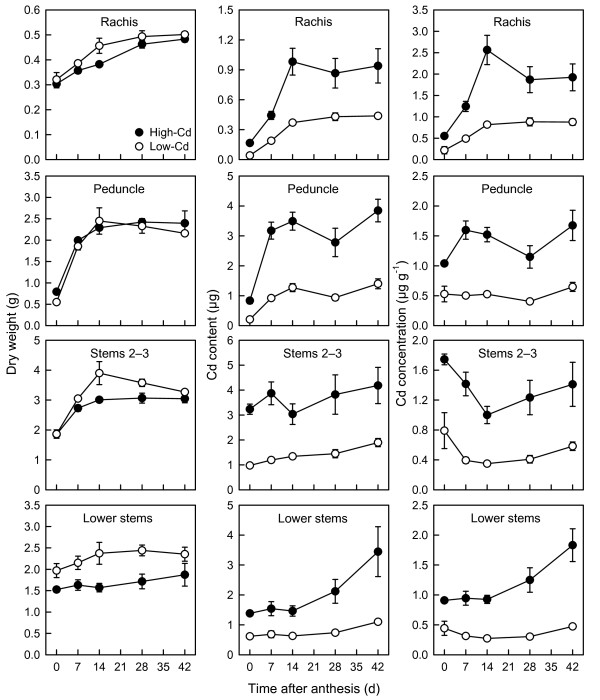
**Growth and cadmium accumulation of stem tissues in durum wheat during grain filling.** Total dry weight, Cd content, and Cd concentration of the rachis, peduncle, stems 2–3, and lower stems of low- (open circles) and high-Cd (closed circles) near-isogenic lines of durum wheat (*Triticum turgidum* subsp. *durum*) between anthesis and 42 d post-anthesis. Plants were grown in chelator-buffered nutrient culture containing 0.5 μM Cd (0.014 nM free activity). Plotted values are means ± SE (*n* = 4 or 5).

Subdivision of shoot tissue (excluding grain) into component parts (Figures 3 and 4) demonstrated that the higher biomass of shoots in the low-Cd NIL was a general feature of biomass accumulation. Except for vegetative tillers (Figure 3) and the peduncle (Figure 4), the biomass of all shoot tissues was greater in low-Cd NIL than in the high-Cd NIL (*P* < 0.05; Figures 3 and 4). The biomass of several shoot tissues increased between anthesis and 7 or 14 DPA as the stem continued to elongate, but were constant thereafter. This was true of flag leaf, peduncle, and stems 2–3 biomass (*P* < 0.05; Figures 3 and 4). Spikelet and rachis biomass increased more consistently throughout the post-anthesis period, whereas the biomass of leaves 2–3, lower leaves, and lower stems did not change over the post-anthesis period (*P* > 0.05). Vegetative tillers, along with grain, accounted for the majority of biomass accumulated by shoots between 0 and 42 DPA, combining for 90% of biomass accumulated during the post-anthesis period (NILs combined). There were no significant ANOVA interactions (*P* > 0.05) between treatments (NIL × harvest) for tissue biomass accumulation during grain filling.

Two patterns of Cd accumulation (both content and concentration) were observed in various shoot tissues (excluding grain) during grain filling. Leafy tissues (spikelets, flag leaf, leaves 2–3, lower leaves, and vegetative tillers) accumulated Cd throughout grain filling (Figure 3), increasing by at least 2-fold. In contrast, stem tissues (rachis, peduncle, stems 2–3, and lower stems) showed a variable pattern of Cd accumulation during grain filling (Figure 4). Accumulation of Cd by the rachis and peduncle increased over the initial 7 (peduncle) or 14 (rachis) DPA (*P* < 0.05) and plateaued thereafter. While stems 2–3 showed negligible change in Cd content, their Cd concentration decreased between 0 and 14 DPA (*P* < 0.05) as the stem continued to elongate. Both the content and concentration of Cd in lower stems were constant during the post-anthesis period, except at the 42 DPA harvest, where both increased (*P* < 0.05). The Cd concentrations of all shoot tissues in the high-Cd NIL were at least 2-fold greater than in low-Cd NIL at all harvests (*P* < 0.001; Figures 3 and 4). With the exception of Cd accumulation in the rachis (both concentration and content), there were no significant ANOVA interactions (*P* > 0.05) between treatments (NIL × harvest) for accumulation of Cd in various tissues during grain filling.

### Whole-plant and grain micronutrient accumulation during grain filling

**Figure 5:**
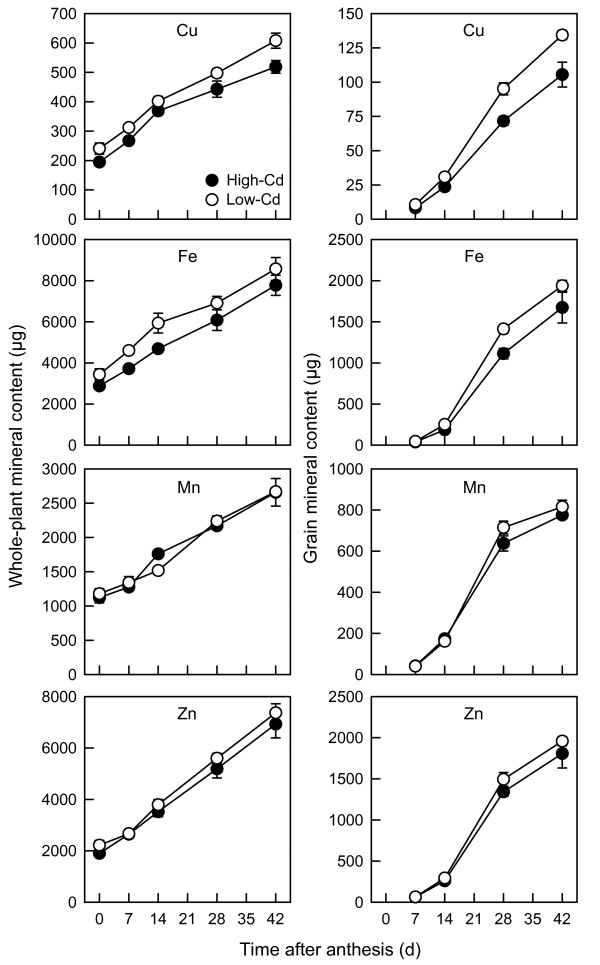
**Whole-plant and grain micronutrient content of durum wheat during grain filling.** Whole-plant and grain Cu, Fe, Mn and Zn content of low- (open circles) and high-Cd (closed circles) near-isogenic lines of durum wheat (*Triticum turgidum* subsp. *durum*) between anthesis and 42 d post-anthesis. Plants were grown in chelator-buffered nutrient culture containing 0.5 μM Cd (0.014 nM free activity). Plotted values are means ± SE (*n* = 4 or 5).

Whole-plant micronutrient content increased by 2.3-fold (Mn), 2.6-fold (Cu, Fe), or 3.5-fold (Zn) between 0 and 42 DPA (NILs combined, *P* < 0.05; Figure 5). Whole-plant Cu and Fe content were higher in the low-Cd NIL (*P* < 0.001), while whole-plant Mn and Zn content were not significantly different between low- and high-Cd NILs (*P* > 0.05). Similar to grain Cd content (Figure 2), grain micronutrient content increased most rapidly between 14 and 28 DPA (Figure 5). However, grain micronutrient content did not significantly differ between low- and high-Cd NILs (Fe and Mn, *P* > 0.05), or was greater (Cu, 30%; Zn, 10%) in the low-Cd NIL (*P* < 0.01). The micronutrient analysis results are fully reported in Additional file 2.

### Cadmium and micronutrient distribution during grain filling

Cadmium, in contrast to the micronutrients (Cu, Fe, Mn, Zn), was retained predominantly in the roots. By the final harvest (42 DPA), accumulation of Cd in the shoots accounted for 13% (low-Cd) or 37% (high-Cd) of whole-plant Cd content (Table 2). In contrast, accumulation of micronutrients occurred primarily in the shoots, ranging between 61% (Cu) and 89% (Mn) of whole-plant accumulation at 42 DPA (Table 2). Furthermore, Cd in grain accounted for 1.3% (low-Cd) or 5% (high-Cd) of whole-plant Cd content, whereas micronutrients in grain was between 20% (Cu) and 31% (Mn) of whole-plant accumulation (Table 2). Similar to shoot Cd concentrations (Figures 3 and 4), the percentage of whole-plant Cd distributed to each shoot tissues was at least 2-fold greater in the high-Cd NIL as compared to low-Cd NIL (*P* < 0.001; Table 2). There were no differences (*P* > 0.05) between the NILs in Cu, Fe, Mn and Zn distribution, with the exception of Mn for rachis and leaves 2–3 (Table 2).

**Table 2:** Whole-plant mineral distribution (%) of durum wheat at grain maturity

**Tissue**	**Cd**^**1**^		**Cu**		**Fe**		**Mn**		**Zn**	
	**Low-Cd**	**High-Cd**	**Low-Cd**	**High-Cd**	**Low-Cd**	**High-Cd**	**Low-Cd**	**High-Cd**	**Low-Cd**	**High-Cd**
Shoots	13 (0.2) *	37 (1.0)	64 (2.4)	61 (0.5)	79 (0.5)	75 (0.9)	89 (1.1)	89 (1.8)	75 (0.8)	74 (0.9)
Grain	1.3 (0.1) *	5.0 (0.4)	22 (0.8)	20 (0.9)	23 (1.2)	21 (1.1)	31 (1.4)	30 (2.5)	27 (0.8)	26 (0.7)
Spikelets	1.1 (0.1) *	3.3 (0.3)	2.6 (0.1)	2.4 (0.1)	5.3 (0.2)	4.9 (0.5)	9.6 (0.6)	8.9 (0.8)	4.1 (0.2)	3.8 (0.1)
Rachis	0.1 (0.0) *	0.2 (0.0)	0.2 (0.0)	0.1 (0.0)	0.2 (0.0)	0.2 (0.0)	1.0 (0.1) *	0.5 (0.1)	0.3 (0.0)	0.3 (0.0)
Peduncle	0.4 (0.0) *	1.0 (0.1)	1.3 (0.1)	1.5 (0.2)	0.4 (0.0)	0.6 (0.2)	1.2 (0.2)	1.3 (0.2)	1.0 (0.1)	1.2 (0.2)
Flag leaf	0.9 (0.1) *	3.2 (0.6)	1.8 (0.1)	1.9 (0.3)	2.0 (0.1)	2.1 (0.3)	1.8 (0.2)	2.0 (0.4)	2.7 (0.1)	2.9 (0.4)
Stems 2–3	0.5 (0.0) *	1.1 (0.0)	1.4 (0.1)	1.5 (0.0)	0.9 (0.0)	0.9 (0.1)	1.2 (0.1)	0.9 (0.1)	1.3 (0.1)	1.2 (0.1)
Leaves 2–3	2.0 (0.1) *	7.0 (0.3)	3.4 (0.3)	3.5 (0.3)	4.3 (0.2)	4.9 (0.5)	5.5 (0.6) *	8.4 (0.5)	4.2 (0.1)	4.0 (0.4)
Lower stems	0.3 (0.0) *	0.9 (0.1)	1.0 (0.1)	1.0 (0.1)	1.4 (0.1)	1.0 (0.1)	0.4 (0.0)	0.3 (0.1)	1.0 (0.1)	0.9 (0.1)
Lower leaves	3.2 (0.3) *	7.8 (1.0)	3.0 (0.5)	2.6 (0.4)	5.4 (0.5)	4.4 (0.5)	18 (2.6)	16 (1.3)	3.6 (0.4)	3.3 (0.6)
Veg. tillers	3.0 (0.2) *	7.7 (0.6)	27 (1.8)	26 (1.0)	36 (1.6)	35 (1.3)	19 (1.7)	21 (0.7)	30 (1.4)	31 (1.2)
Roots	87 (0.2) *	63 (1.0)	36 (2.4)	39 (0.5)	21 (0.5)	25 (0.9)	11 (1.1)	11 (1.8)	25 (0.8)	26 (0.9)

^1^ Mineral content (Cd, Cu, Fe, Mn, Zn) of low- and high-Cd near-isogenic lines of durum wheat (*Triticum turgidum* subsp. *durum*) at physiological grain maturity (42 d post-anthesis) expressed as a percentage of the whole-plant content. Plants were grown in chelator-buffered nutrient culture containing 0.5 μM Cd (0.014 nM free activity). Significant differences between near-isogenic lines as determined by *F*-test-protected Student’s *t*-test are indicated by * (*P* ≤ 0.05). The *t*-tests were computed only when the ANOVA of the complete data set (all harvests for each tissue) yielded significant NIL or NIL × harvest treatment effects. Numbers in parenthesis are SE (*n* = 4).

Although the amount of Cd accumulated in shoot tissues differed between the low- and high-Cd NILs, the relative pattern of Cd accumulation in the shoots was similar (Table 2). The largest pools of Cd in the shoots of both NILs were the grain and leafy tissues (spikelets, flag leaf, leaves 2–3, lower leaves, and vegetative tillers). Stem tissues (rachis, peduncle, stems 2–3, and lower stems) accumulated little Cd, individually accounting for ≤1% of whole-plant Cd content in both NILs. The Cd content in leaves of both NILs was associated with leaf position or age; accumulation was highest in the oldest leaves (lower leaves) and lowest in the youngest leaves (flag leaf) (Table 2; the same pattern also shown for leaf Cd concentration, Figure 3).

Uptake of Cd by the roots and export of Cd from the roots to the shoots occurred throughout grain filling in both the low- and high-Cd NILs, as shown by increasing whole-plant, shoot, and root Cd content (Figure 1). However, there was no indication of net remobilization of Cd from shoot tissues during grain filling (Table 3). Most shoot tissues accumulated Cd throughout grain filling (e.g. spikelets, flag leaf), reaching maximum Cd content at the final harvest (42 DPA). The rachis and peduncle accumulated Cd between 0 and 14 DPA and plateaued thereafter. In contrast, the micronutrient content (Cu, Fe, Mn, Zn) of tissues near the grain decreased markedly between 14 and 42 DPA (Table 3). The Cu content of the spikelets, rachis, and flag leaf decreased by approximately 50% after 14 DPA. Less Cu (20-40%) was remobilized from the peduncle, leaves 2–3, and lower leaves. Manganese was remobilized (50-70%) from the peduncle, flag leaf, stem 2–3, and lower stems. The majority of Fe (70-80%) and Zn (50-70%) was remobilized from the upper stem (rachis, peduncle, and stem 2–3) after 14 DPA. Iron was remobilized to a lesser extent (20-50%) from the flag leaf, leaves 2–3, and lower stems, while Zn was not remobilized from the leaves.

**Table 3:** Remobilization of biomass and minerals in durum wheat during grain filling

		**Tissue dry weight and mineral content (% of maximum, low-Cd:high-Cd)**^**1**^												
	**Harvest (DPA**^**2**^**)**	**Grain**	**Spikelets**	**Rachis**	**Peduncle**	**Flag leaf**	**Stems 2–3**	**Leaves 2–3**	**Lower stems**	**Lower leaves**	**Veg. tillers**	**Shoots**	**Roots**	**Total**
DW	0	NA^3^	57:63	64:63	23:33	84:80	48:60	89:83	81:81	100:91	NA	23:21	64:57	33:31
	7	3:4	83:76	77:74	76:82	97:94	78:89	97:93	88:87	86:87	4:3	30:28	81:70	43:39
	14	15:17	88:80	91:79	100:95	100:96	100:98	95:90	97:84	87:89	28:33	46:46	87:83	57:56
	28	75:77	90:91	98:96	95:100	98:100	92:100	97:99	100:92	75:89	54:62	73:76	93:91	78:80
	42	100:100	100:100	100:100	88:99	100:93	84:99	100:100	96:100	84:100	100:100	100:100	100:100	100:100
Cd	0	NA	4:4	9:17	15:22	21:17	51:77	21:20	56:40	45:28	NA	20:15	35:26	33:22
	7	3:1	12:9	43:45	66:83	37:28	63:92	35:29	62:45	51:37	3:2	29:23	45:43	43:36
	14	12:8	24:21	85:100	91:91	52:37	71:73	44:38	57:42	66:41	22:25	43:33	60:53	58:46
	28	79:70	56:55	98:88	67:72	73:74	76:91	69:69	67:62	73:70	49:56	66:66	91:82	88:76
	42	100:100	100:100	100:96	100:100	100:100	100:100	100:100	100:100	100:100	100:100	100:100	100:100	100:100
Cu	0	NA	63:77	55:72	31:41	82:83	92:91	92:89	93:91	100:100	NA	30:29	57:51	40:38
	7	8:8	100:100	99:100	78:100	100:100	93:100	100:100	100:100	85:91	5:5	40:39	72:70	51:51
	14	23:22	97:89	100:73	100:94	99:98	100:82	94:93	100:100	80:88	38:44	58:58	80:91	66:71
	28	71:68	66:73	46:53	60:71	79:82	65:63	79:88	82:81	56:73	53:63	72:77	99:99	82:85
	42	100:100	57:55	43:45	64:72	53:54	90:98	66:70	90:86	59:80	100:100	100:100	100:100	100:100
Fe	0	NA	31:39	25:41	14:30	56:64	57:63	70:77	76:77	90:88	NA	24:22	86:83	40:37
	7	2:2	65:70	83:92	61:87	86:99	96:100	95:100	100:100	98:100	5:4	37:35	97:89	54:48
	14	13:11	79:80	100:100	100:100	100:100	100:74	100:98	93:95	100:94	31:32	56:50	100:92	69:60
	28	73:67	93:97	25:40	37:48	71:78	36:42	83:92	64:83	75:93	49:56	71:73	99:95	81:78
	42	100:100	100:100	14:25	17:29	50:53	25:32	70:83	68:68	81:99	100:100	100:100	85:100	100:100
Mn	0	NA	24:34	17:41	19:32	83:80	89:100	80:82	100:100	89:80	NA	37:37	87:79	44:42
	7	5:5	43:52	40:44	96:100	100:100	100:88	100:100	73:59	83:84	3:2	45:46	81:58	50:48
	14	20:22	63:83	72:93	100:68	90:92	62:50	75:99	40:52	86:90	25:43	54:63	71:86	57:66
	28	88:82	53:60	100:100	40:29	62:58	47:27	80:87	25:30	81:88	61:59	80:78	100:100	84:82
	42	100:100	100:100	98:77	57:44	38:42	50:37	76:87	26:31	100:100	100:100	100:100	84:93	100:100
Zn	0	NA	52:67	26:38	16:26	65:71	70:77	73:77	100:94	90:66	NA	20:20	61:49	30:27
	7	3:3	77:78	62:57	65:79	80:86	91:100	80:83	100:100	76:59	4:4	28:28	61:68	36:38
	14	15:14	90:77	100:78	100:100	100:91	100:78	93:82	84:74	91:60	29:34	47:44	65:71	51:51
	28	76:74	59:76	88:100	35:40	86:99	46:45	85:98	70:73	67:66	52:61	68:73	100:80	76:75
	42	100:100	100:100	53:49	31:32	97:100	36:36	100:100	83:90	100:100	100:100	100:100	100:100	100:100

^1^ Dry weight (DW) and mineral content (Cd, Cu, Fe, Mn, Zn) of low- and high-Cd near-isogenic lines of durum wheat (*Triticum turgidum* subsp. *durum*) during grain filling expressed at each harvest as a percentage of the maximum accumulation at any harvest (low-Cd:high-Cd). ^2^*DPA* days post-anthesis. ^3^*NA* not applicable.

## Discussion

We have used chelator-buffered hydroponic culture to mimic the low activities of trace elements present in uncontaminated agricultural soils [31]. The supply of nutrients was sufficient for healthy plant growth and grain development. Plants grew vigorously and without visual symptoms of Cd phytotoxicity. Micronutrient concentrations in mature grain were similar (Cu, Mn) or 2 to 3-fold greater (Fe, Zn) than plants grown under field conditions [5]. Higher concentrations of micronutrients might be expected given the unrestricted transpirational flow inherent to hydroponic culture. High transpirational flow should also favour movement of Cd to the grain. Concentrations of Cd in mature grain (Figure 2D) were 2 to 4-fold greater than observed in the same NILs grown in the field over 11 site-years [5], and comparable to concentrations in a pair of related NILs grown in chelator-buffered culture [22]. We conclude that the chelator-buffered hydroponic culture provided a reasonable approximation of Cd and micronutrient accumulation under field conditions.

Cadmium uptake and distribution presumably reflects a complex array of factors operating at the cellular, tissue, and whole plant level, as affected by genotype and environment. Some of these factors are associated with *Cdu1*, the major QTL for accumulation of Cd in durum wheat grain [9-11]. Other factors are independent of *Cdu1*, and result in a pattern of Cd accumulation that is common to both near-isogenic lines. Since the NILs share a pedigree with the majority of modern Canadian durum wheat cultivars [32], these common patterns should be indicative of Cd accumulation by durum wheat more generally.

The most important difference between the low- and high-Cd NILs is the degree of root-to-shoot Cd translocation [19]. Restricted Cd translocation is also the primary determinant of intraspecific variation in Cd accumulation in rice grain [3,33]. In our study, the majority of Cd absorbed by plants was retained in the roots, and significantly more was retained by roots of the low-Cd NIL (Figure 1). This difference was apparent in seedlings (Table 1), confirming previous results [19,20,22,23], and persisted throughout grain filling. At grain maturity, accumulation of Cd in the shoots was 13% (low-Cd) or 37% (high-Cd) of whole-plant Cd accumulation (Table 2). Differences in root-to-shoot partitioning of Cd were evident throughout the shoot: accumulation of Cd in every shoot tissue was at least 2-fold higher in the high-Cd NIL at all harvests (Figure 3 and 4), a pattern of accumulation that is maintained under field conditions (Harris and Taylor, unpublished data). By 7 DPA, when less than 5% of final grain DW had accumulated (Table 3), concentrations of Cd in the grain were already 2-fold greater in the high-Cd NIL. This was true despite the fact that the low- and high-Cd NILs did not differ in whole-plant Cd uptake (Figure 1), indicating no significant difference in absorption of Cd by roots. Earlier studies with wheat have shown that genotypic differences in grain Cd accumulation were unrelated to short-term Cd uptake by roots [19,21,23,25].

Field studies have demonstrated that concentrations of micronutrients (Cu, Fe, Mn and Zn) in grain are typically not affected by the low-Cd trait [5]. Our results confirm that the low-Cd trait has no detrimental effect on micronutrient accumulation (Figure 5) or the distribution of micronutrients between tissues (Table 2). Thus, breeding for low Cd should not negatively affect the micronutrient content of durum wheat grain [7].

Although the NILs differed significantly in the magnitude of Cd accumulation in shoot tissues, the pattern of Cd accumulation was remarkably similar in both lines (Table 2), an observation that is most clearly illustrated in the animated time-course of Cd accumulation (Additional file 2: mini-website). Both NILs absorbed Cd from the nutrient solution and transported Cd to the shoots throughout grain filling (Figure 1). Although some reports suggest that uptake of Cd by roots and root-to-shoot translocation are limited during grain filling [21,29], others suggest that both rice and wheat continue to absorb Cd from the growth medium and export it to the shoots. Rodda and Reid [30] showed that 40% of Cd in mature grain could be accounted for by absorption and translocation of Cd during grain filling in rice grown in hydroponic culture. Similarly, the shoot Cd content of rice grown in Cd-contaminated soil increased by a third between early grain filling and grain maturity [27]. Measurable quantities of Cd in xylem exudates collected during early grain filling confirmed root-to-shoot Cd transport [27]. Hart *et al.*[22] reported that the Cd concentration of the flag leaf and spike of durum wheat grown in chelator-buffered culture increased throughout grain filling, which is consistent with our results (Figure 3).

Cadmium accumulated in the shoot tissues (especially the leaves) in a declining gradient towards the head (Figure 3; Table 2), a pattern that is typical of cereals [20,27,28]. Leafy tissues (spikelets, leaves, vegetative tillers), which are terminal sinks for transpirational flow, accumulated Cd throughout grain filling. In contrast, the Cd content of stem tissues were generally constant during grain filling, except for when they increased in weight and Cd content during early grain filling (Figure 4). Although the stems were subject to high Cd flow towards the grain and leaves, they were nonetheless minor pools of Cd (individually <1% of whole-plant Cd content; Table 2). Collectively, these results show that the relative size and temporal development of shoot Cd pools varies between shoot organs, highlighting the need to quantify these pools when studying Cd transport to grain.

The timing of Cd accumulation in grain was strongly correlated with grain biomass (DW) accumulation in both NILs. Like rice [30], it was not constant throughout grain filling. When stems continued to elongate during early grain filling (<14 DPA), grain DW and Cd both accumulated slowly (Figure 2). The highest rate of grain filling was between 14 and 28 DPA, a period in which both NILs accumulated 60% of total grain DW and 61-66% of total grain Cd content.

Remobilization of Cd stored in the leaves, particularly the flag leaf, has been suggested as an important source of Cd transported to the grain in cereals [20,21,27-29]. While our data do not preclude the movement of Cd from leaves, there was no indication of net remobilization during grain filling (Table 3). Thus the rate of Cd remobilization from leaf Cd pools, either from pre-existing symplastic Cd pools or from apoplastic Cd accumulated after xylem unloading, must be less than the rate of import to the leaves via the xylem.

Several observations support the conclusion that little Cd is exported from the leaves during grain filling and, as a result, leaf Cd pools are minor contributors to grain Cd accumulation. The first observation is that our experimental system is capable of detecting net remobilization during grain filling, but we have not observed net remobilization of Cd. Even though uptake and translocation of Cu, Fe, Mn, and Zn to the shoots continued throughout grain filling (Figure 5), and the majority of absorbed micronutrients were transported to the shoots (61 to 89%, Table 2), we were still able to detect substantial remobilization of micronutrients from shoot tissues near the grain (Table 3). Between 40 and 80% of Cu and Fe were remobilized from the flag leaf and upper stem after 14 DPA, and 60-70% of Zn was remobilized from the peduncle and stem 2–3. Even Mn, which is typically considered to have low mobility in the phloem [34-36], was strongly remobilized from the stem and flag leaf (50-70% export).

The second observation is related to the fact that low- and high-Cd NILs differ not only in root-to-shoot Cd translocation, but also in Cd transport from the flag leaf to the grain, which we have previously shown to be approximately 2-fold greater in the high-Cd NIL [37]. If the flag leaf was a primary source of Cd transported to the grain, it might be expected that greater transport would lead to lower Cd content in the flag leaf of the high-Cd NIL, or at a minimum the flag leaf Cd content of NILs would converge during grain filling. The size of the Cd pool in the flag leaf leads to the conclusion that a majority of this Cd pool would need to be remobilized to the grain if the flag leaf was the leading contributor to Cd accumulation in grain. In fact, the entire pool of Cd accumulated post-anthesis by the flag leaf would only account for 50% (high-Cd NIL) or 56% (low-Cd NIL) of Cd accumulated in the grain. Our results show that Cd accumulation in the flag leaf was more than 2-fold greater in the high-Cd NIL at all harvests (Figure 3).

The lack of net remobilization of Cd from leaves during grain filling (Table 3) is consistent with previous studies that documented limited movement of Cd from leaves via the phloem, but does not imply that Cd transport via the phloem is unimportant to Cd accumulation in the grain. To be sure, there is evidence for Cd transport via the phloem [35-40], including direct measurement of Cd in phloem sap [27,41,42]. However, extensive evidence documenting low rates of remobilization of Cd from leaves towards growing sink tissues, such as grain, roots, and young expanding leaves [35,36,39,40], suggests that Cd has limited phloem mobility in wheat. As an example, the low- and high-Cd NILs have been shown to differ in ^109^Cd transport from the flag leaf to the grain via the phloem [37], but the majority (75-80%) of absorbed ^109^Cd was retained in the labelling region; only 10 to 15% of the ^109^Cd was transported to the grain after 7 d. In contrast, 65-70% of ^65^Zn absorbed by the flag leaf was transported to the grain [37]. Similarly, in seedlings of *Triticum* species, ^109^Cd applied to the mature first leaf was largely (~95%) retained in the labelled leaf; between 1 and 2% of the absorbed ^109^Cd was transported to other shoot tissues after 42 h [39,40]. Transport of phloem-mobile ^86^Rb to other shoot tissues was 10-fold higher than that of ^109^Cd [40]. Several other studies have also documented evidence of low rates of remobilization of Cd from leaves [35,36,43].

Notwithstanding these low rates of remobilization, transport via the phloem is important to Cd accumulation in grain. This must be true given the xylem discontinuity present in the grain pedicel of wheat [44]; Cd transport to the grain is ultimately dependent on delivery through the phloem. In this regard, a key question is: what is the origin of Cd loaded into the phloem that is transported to the grain? Is it Cd that essentially flows uninterrupted from the roots through the stem on its way to the grain, or a genuine remobilization of Cd that has been deposited in leaf tissues? In our study, roots continued to absorb Cd from the nutrient solution and export Cd to the shoot throughout the grain filling period, as shown by increasing whole-plant and shoot Cd accumulation. This result, combined with the absence of net remobilization of Cd from leaves, strongly suggests that the majority of Cd accumulated in the grain was transported from the roots through the stems to the grain. Recently, real-time imaging of positron-emitting ^107^Cd tracer in rice plants [45,46] identified stem nodes as important sites of direct xylem-to-phloem Cd transfer. Accumulation of ^107^Cd in short-term experiments was localized principally in the stems, concentrated at nodes and grain, but little accumulation occurred in the leaves [45,46]. Tissue-level imaging of stems provided evidence for intervascular Cd trafficking at nodes [47,48]. Studies using detached wheat shoots labelled with ^109^Cd below the flag leaf node leads to similar conclusions. Disrupting the phloem by steam-girdling the upper peduncle decreased Cd accumulation in the grain by 3-fold and increased Cd retention in the peduncle [35,38], indicating that the majority of ^109^Cd transported to the grain had been transferred from the xylem to the phloem in the lower peduncle and/or flag leaf node.

Although stem tissues accumulated little Cd (Table 2), they are likely important sites for xylem-to-phloem Cd transfer, a necessary step in Cd transport to the grain. Such flow-through movement of Cd from roots through the stems to the grain could be affected by the conditions under which Cd is supplied to the plant. Our results shows that when root-to-shoot Cd flux is continuous (such as in solution culture) the majority of Cd in the transpiration stream is delivered to high transpiration tissues (spikelets, leaves, and vegetative tillers). Cadmium accumulation in these tissues is passive, reflecting the degree to which Cd is released by the roots, which is lower in the low-Cd NIL. While translocation of Cd is driven by transpirational flow, and treatments that restrict transpiration reduce transport of Cd to the shoots [14,49], variation in transpiration rates do not explain genotypic differences in shoot and grain Cd accumulation of cereals [14,50,51], including for the NILs used in this study [52]. When Cd is less abundant in the transpiration stream it might be readily removed from the xylem and transported to the grain via the phloem. Following application of trace quantities (250 pM) of ^109^Cd to the fourth stem-internode of intact durum wheat, ^109^Cd accumulated in the stem in a declining gradient towards the spike [37]. Subsequently, ^109^Cd was remobilized from the stem and transported to the grain; very little ^109^Cd accumulated in the spikelets or leaves [37]. Similarly, Cd applied to intact wheat plants via a stem flap cut below the flag leaf node initially accumulated in the peduncle and flag leaf, but was later remobilized from the peduncle, but not the flag leaf, to the grain [38].

The mechanistic basis for restricted root-to-shoot Cd translocation in low-Cd genotypes of durum wheat remains to be determined. Cadmium transport in plants has been shown to be regulated by a variety of transport proteins that mediate uptake from the rhizosphere, symplastic sequestration, xylem loading, xylem-to-phloem transfer, and remobilization via the phloem [3,33]. Notwithstanding this complexity, studies of inheritance of the low Cd trait in durum wheat have shown that grain Cd concentration is probably controlled by a single, dominant gene [53]. It is difficult to reconcile these observations unless a single gene was involved in a cellular phenomenon that is pathway-independent. Apoplastic or symplastic loading of the phloem in the leaves or stems, followed by unloading in sink tissues (e.g. grain) is fundamentally different from xylem loading in roots, which simply requires efflux from xylem parenchyma into the lumen of xylem vessels. Recently, allelic variation in a rice P_1B_-ATPase, *OsHMA3*, was associated with major QTLs for Cd accumulation in grain [54,55]. OsHMA3 is a Cd-specific transporter that is localized to the tonoplast [54,55]; it mediates the sequestration of Cd in the vacuole and restricts root-to-shoot translocation of Cd [54]. Loss-of-function mutations in *OsHMA3* observed in some cultivars resulted in elevated Cd accumulation in shoots and grain [54-56]. Arabidopsis HMA3 (AtHMA3) also localizes to the tonoplast [57]. Recently, a screen of a world-wide collection of accessions identified *AtHMA3* as the primary determinant of natural variation in leaf Cd [58]. The transport activity of HMA3 is a process that is pathway independent. A wheat homolog of *HMA3* could contribute to sequestration of Cd in root cell vacuoles, limiting radial Cd transport to the stele and subsequent xylem loading. Similarly, sequestration of Cd in the vacuole of mesophyll or bundle sheath cells could restrict phloem loading in the leaves. A major QTL (designated as *Cdu1*) controlling accumulation of Cd in grain of durum wheat has been reported on chromosome 5B [9]. Fine mapping has localized *Cdu1* to a 0.7 cM interval that explains more than 80% of the variation in Cd accumulation in grain [10]. However, the regions collinear to *Cdu1* on rice chromosome 3 and *Brachypodium distachyon* chromosome 1 contain no candidate genes with putative metal transporter activity [10]. Breaks in microcollinearity were also documented in this region [10], indicating that map-based cloning will be required to isolate the gene responsible for *Cdu1* in durum wheat.

## Conclusions

Genotypic variation in the accumulation of Cd in grain of durum wheat is attributable to *Cdu1*, a major QTL controlling accumulation of Cd in grain [9-11]. Our results show that *Cdu1* controls accumulation of Cd in grain by regulating root-to-shoot translocation of Cd. Differences between NILs in root-to-shoot partitioning of Cd were evident in all shoot tissues (including grain) and Cd accumulation was at least 2-fold higher in the high-Cd NIL at all harvests. Continued Cd uptake and the absence of net remobilization of Cd from leaves during grain filling supports a direct pathway of Cd transport from roots to grain via xylem-to-phloem transfer in the stem. As the roots retained the majority of absorbed Cd, variation in translocation of this Cd pool greatly affects Cd levels in shoots and grain. Identification of the genetic factor(s) responsible for *Cdu1* will enable selection of low-Cd genotypes from durum wheat germplasm with different genetic backgrounds without phenotyping or revalidating the marker-*Cdu1* allele relationship [12].

## Methods

### Plant growth

A pair of near-isogenic lines (NILs) of durum wheat differing in accumulation of Cd in grain [59], were used in these experiments. Field studies have shown that the high-Cd line (8982-TL-H) accumulates 2.5-fold greater Cd concentrations in mature grain than the low-Cd line (8982-TL-L) [5].

Seeds were surface sterilised in 1.2% NaOCl for 20 min, rinsed, and imbibed for 24 h in an aerated solution of 1 mM CaCl_2_ and 5 mg L^-1^ Vitavax fungicide (Uniroyal Chemical Ltd, Calgary, AB, Canada). The germinated seeds were placed on nylon mesh suspended over 10 L of aerated, chelator-buffered nutrient solution. The nutrient solution was prepared in reverse osmosis (RO) water (<3 μS cm^-1^) and contained 1.0 mM Ca(NO_3_)_2_, 0.3 mM Mg(NO_3_)_2_, 0.3 mM NH_4_NO_3_, 0.25 mM KNO_3_, 0.1 mM K_2_HPO_4_, 0.1 mM K_2_SO_4_, 50 μM KCl, 100 μM Fe(NO_3_)_3_, 10 μM H_3_BO_3_, 0.2 μM Na_2_MoO_4_, 10 μM ZnSO_4_, 2 μM CuSO_4_, 1 μM MnSO_4_, 0.5 μM CdCl_2_, 0.1 μM NiCl_2_, 138.6 μM N-(2-hydroxyethyl)ethylenediaminetriacetic acid (HEDTA), 1.42 mM KOH, and 2 mM 2-(N-morpholino)ethanesulfonic acid (MES) buffer (pH 6.0). The supplied concentration of HEDTA provided a 25 μM excess over the total concentration of transition metal cations, thereby buffering free metal activities at environmentally relevant levels [31]. Free ion activities (p(M^n+^)) were 10.84 (Cd^2+^), 13.44 (Cu^2+^), 16.64 (Fe^3+^), 7.75 (Mn^2+^), 14.44 (Ni^2+^), and 9.94 (Zn^2+^) as calculated by GEOCHEM-PC [60].

Seedlings were grown for 3 days in the dark, and then a further 4 days in a controlled environment growth chamber (16 h daylight, 21/16°C day/night temperature, and 450 μmol m^-2^ s^-1^ photosynthetically active radiation). Caryopses were removed after 7 d and the seedlings were transferred to 10-L polyethylene buckets (under the same growth conditions) containing aerated, chelator-buffered nutrient solution as described above. Each bucket held two seedlings, supported independently by polyethylene mesh baskets mounted in opaque polycarbonate lids. Buckets containing nutrient solutions were suspended in a common water bath to limit temperature fluctuations and maintain a consistent root temperature in all experimental containers. Nutrient solutions were replaced every 14 d. Between solution changes, RO water was added to maintain a constant solution volume, and solution pH and electrical conductivity (EC) were monitored daily. The pH was adjusted with 1.25 N HNO_3_ or KOH when it deviated from 6.0 ± 0.1.

Electrical conductivity (EC) was used to estimate depletion of nutrient solutions on a daily basis. Equal volumes of two daily addition stock solutions were added to adjust the solution EC to the nominal level (580 μS cm^-1^). The amount of daily addition solution required was determined by titration of addition volume against EC. The composition of daily addition stock solutions (Stock 1: 40 mM KH_2_PO_4_, 210 mM NH_4_NO_3_, 420 mM KNO_4_, 20 mM (NH_4_)_2_SO_4_; Stock 2: 20 mM Ca(NO_3_)_2_, 40 mM Mg(NO_3_)_2_, 0.4 mM H_3_BO_3_) was optimized in preliminary experiments to maintain shoot nutritional status and to minimize solution pH fluctuation. The maximum rate of nutrient usage occurred from 14 d pre-anthesis to 7 d post-anthesis. During this period the mean daily decline in solution EC was 60 μS cm^-1^, corresponding to a daily addition of 150 μM NH_4_^+^, 450 μM NO_3_^2-^, 24 μM PO_4_^3-^, 12 μM SO_4_^2-^, 276 μM K^+^, 12 μM Ca^2+^, 24 μM Mg^2+^, and 0.24 μM H_3_BO_3_.

High nutrient concentrations inherent to solution culture can induce high rates of tillering in cereal species [22,61,62], resulting in uneven grain maturation [61]. In preliminary experiments where plants were allowed to grow unchecked, each plant produced 30–40 tillers that initiated flowering over 2 to 3 weeks. In order to relate whole-plant Cd accumulation and partitioning to grain maturation, only the first four tillers (main stem and three auxiliary tillers) were allowed to develop (the coleoptile tiller, if present, was always removed). Additional tillers were removed every 2–3 d, beginning after the first solution change (15 d post-transplantation) and continuing until anthesis. All pre-anthesis tillers were discarded. After anthesis, tillers were allowed to develop for up to 7 days. This prevented tillers that developed post-anthesis from flowering. These tillers were collected and pooled at harvest (designated vegetative tillers).

As flowering heads (4 per plant) began to emerge, they were monitored daily and tagged at the initiation of anthesis (anther protrusion). Plants were harvested at anthesis, and at 7, 14, 28 and 42 d post-anthesis (DPA). In preliminary experiments, physiological grain maturity (maximum grain dry weight) was achieved between 35 and 42 DPA. To ensure that harvests were completed at a uniform developmental stage, each plant was harvest at the designated number of days after the date of first flowering. Thus, replicate plants within a harvest were not necessarily harvested on the same day; most were spread over 6 to 8 d (range: 3–11 d). Flowering of all four tillers within each plant was initiated within 2 to 4 d of each other.

### Plant sampling and analysis

Immediately prior to the first solution change (14 d post-transplantation), five randomly selected buckets of each NIL were harvested (21-d-old seedlings). The plants in these buckets were replaced during the first solution change with one plant from an adjacent bucket. The remaining buckets were thinned to one plant per bucket. Thereafter, plants were randomly assigned to harvests, and the position of the buckets were re-randomized every 14 d. At each harvest, plants were separated into grain, spikelets (glumes, palea, lemma, and rachilla), rachis, peduncle, flag leaf, stems 2–3, leaves 2–3, lower stems, lower leaves, vegetative tillers, and roots. Leaves (lamina and sheath) and stems were labelled from the top of the plant. The flag leaf and peduncle are the first leaf and first stem internode below the spike. Leaves 2–3 and stems 2–3 are the combined second and third leaves and stem internodes. Lower leaves and lower stems are all the remaining leaves and stems. Vegetative tillers included tillers that developed between anthesis and harvest.

Shoot tissues were washed immediately on harvest in RO water for 30 s, while the roots were triple rinsed (RO water, 1 min; 1 mM CaCl_2_, 5 min; RO water, 1 min) and blotted dry. Plant samples were oven-dried at 65°C for 3 d, weighed, and finely ground in a stainless steel mill. Ground sub-samples (0.5 g) were digested at 95°C in solutions containing 5 mL of trace-metal grade, concentrated HNO_3_ and 2 mL of 30% H_2_O_2_, and diluted to 50 mL with deionized water (>18 MΩ purity). Cadmium and Cu were determined by graphite furnace atomic absorption spectroscopy (AAS), and Fe, Mn, and Zn were determined by flame AAS (AAnalyst 700; PerkinElmer, Waltham, MA). Reagent blanks and a NIST Standard Reference Material (NIST No. 8436 durum wheat flour) were included in each batch of samples for quality control. Recovery of the reference concentration values were (mean ± SD) 98 ± 9% (Cd), 94 ± 4% (Cu), 102 ± 6% (Fe), 101 ± 3% (Mn), and 105 ± 4% (Zn).

The experiment was arranged in a completely randomized design with unequal replication. All harvests had five replicates except for the final harvest (42 DPA), which due to space constraints had four replicates. Data were analysed by analysis of variance. When significant (*P* ≤ 0.05) treatment effects were detected, differences between treatment means were determined by Student’s *t*-test or Tukey’s test for pairwise and multiple comparisons, respectively. Data for some variables were log_e_ transformed to attain homogeneity of variance. The entire experiment as described above was repeated (with similar results) to ensure repeatability of results.

Complete growth and metal accumulation data collected during grain filling are presented as an interactive website (Additional file 2: Mini-website showing Cd, Cu, Fe, Mn, and Zn accumulation in low- and high-Cd near-isogenic lines of durum wheat during grain filling).

## Competing interests

The authors declare no competing interests.

## Authors’ contributions

NSH and GJT contributed equally to the conception and design of the study,and to preparation of the manuscript. NSH completed the experiments and analyses. Both authors read and approved the final manuscript.

## Supplementary Material

Additional file 1 Micronutrient accumulation by durum wheat seedlings. Click here for file

Additional file 2 Mini-website showing Cd, Cu, Fe, Mn, and Zn accumulation in low- and high-Cd near-isogenic lines of durum wheat during grain filling. Click here for file
