# Supplementary material for: Cadmium uptake and partitioning in durum wheat during grain filling
Source: BMC Plant Biol. 2013 Jul 16;13:103. doi: 10.1186/1471-2229-13-103 (PMC3726410; doi:10.1186/1471-2229-13-103)
Supplement: Additional file 1 — Micronutrient accumulation by durum wheat seedlings. [file 1471-2229-13-103-S1.pdf]

Additional file 1: Micronutrient accumulation by durum wheat seedlings.
Micronutrient content (µg plant^{-1}) of seedlings of low- and high-Cd near-isogenic lines of durum wheat (*Triticum turgidum* subsp. *durum*) grown for 21 d in chelator-buffered nutrient culture containing 0.5 µM Cd (0.014 nM free activity).

	Low-Cd	High-Cd
Cu		
Whole plant	3.4 (0.2)	3.2 (0.2)
Shoot	2.5 (0.1)	2.3 (0.1)
Root	0.9 (0.1)	0.9 (0.1)
Fe		
Whole plant	55.0 (2.3)	53.7 (3.2)
Shoot	39.5 (1.9)	38.5 (2.4)
Root	15.5 (0.5)	15.2 (1.0)
Mn		
Whole plant	46.2 (2.1)	51.8 (2.2)
Shoot	38.3 (1.9)	44.0 (1.9)
Root	7.9 (0.6)	7.8 (0.7)
Zn		
Whole plant	29.8 (0.9)	30.0 (1.5)
Shoot	20.9 (0.6)	20.8 (0.9)
Root	9.0 (0.5)	9.3 (0.8)

There were no significant differences (P>0.05) between near-isogenic lines for any of the variates as determined by ANOVA (F-test). Numbers in parenthesis are SEM (n = 5).
