# Supplementary material for: Cadmium uptake and partitioning in durum wheat during grain filling
Source: BMC Plant Biol. 2013 Jul 16;13:103. doi: 10.1186/1471-2229-13-103 (PMC3726410; doi:10.1186/1471-2229-13-103)
Supplement: Additional file 2 — Mini-website showing Cd, Cu, Fe, Mn, and Zn accumulation in low- and high-Cd near-isogenic lines of durum wheat during grain filling. [file 1471-2229-13-103-S2.zip › fe_anim.html]

Harris and Taylor 2013: Animated iron accumulation


# Cadmium uptake and partitioning in durum wheat during grain filling

## Harris NS and Taylor GJ (2013) *BMC Plant Biology* 13:103

### Site menu:

- Introduction
- Cadmium
- Copper
- Iron
- Manganese
- Zinc

  

### Page contents:

Interactive tissue map | 
Animated iron accumulation

## Animated iron accumulation

  

Iron accumulation (µg) between anthesis and physiological grain maturity (42 d post-anthesis)
of low- and high-Cd near-isogenic lines of durum wheat. Plotted values are means ± SE (n=4 or 5).

©2013 Neil S. Harris & Gregory J. Taylor
