# Supplementary material for: Cadmium uptake and partitioning in durum wheat during grain filling
Source: BMC Plant Biol. 2013 Jul 16;13:103. doi: 10.1186/1471-2229-13-103 (PMC3726410; doi:10.1186/1471-2229-13-103)
Supplement: Additional file 2 — Mini-website showing Cd, Cu, Fe, Mn, and Zn accumulation in low- and high-Cd near-isogenic lines of durum wheat during grain filling. [file 1471-2229-13-103-S2.zip › index.html]

Harris and Taylor 2013: Cadmium uptake and partitioning in durum wheat during grain filling


# Cadmium uptake and partitioning in durum wheat during grain filling

## Harris NS and Taylor GJ (2013) *BMC Plant Biology* 13:103

### Site menu:

- Introduction
- Cadmium
- Copper
- Iron
- Manganese
- Zinc

  

### Page contents:

## Background

This self-contained mini-website contains the complete growth and metal (Cd, Cu, Fe, Mn, Zn) accumulation data
collected between anthesis and physiological grain maturity (42 days post-anthesis, DPA) for low- and high-Cd
near-isogenic lines of durum wheat (*Triticum turgidum* subsp. *durum*) grown in chelator-buffered nutrient culture.

The results are presented as: *i)* an interactive tissue map, and *ii)* animated time-course of metal accumulation.

Cadmium
Interactive tissue map
Animated cadmium accumulation

Copper
Interactive tissue map
Animated copper accumulation

Iron
Interactive tissue map
Animated iron accumulation

Manganese
Interactive tissue map
Animated manganese accumulation

Zinc
Interactive tissue map
Animated zinc accumulation

  

### Durum wheat growth movies

Time-lapse of durum wheat growth

[![


The browser does not support the video content  
Download video: MP4

](flash/start_0201_cbns.jpg)](flash/0201_cbns.mp4)

Time-lapse of durum wheat growth. Plants of low- and high-Cd near-isogenic lines of durum wheat were grown from seedlings to physiological grain maturity in chelator-buffered nutrient culture.  
Download video: MP4

Durum wheat plant, 14 DPA

[![


The browser does not support the video content  
Download video: MP4

](flash/start_plant_14dpa.jpg)](flash/plant_14dpa.mp4)

Durum wheat plant at 14 days post-anthesis grown in chelator-buffered nutrient culture.  
Download video: MP4

©2013 Neil S. Harris & Gregory J. Taylor
